# Risk stratification of cardiac metastases using late gadolinium enhancement cardiovascular magnetic resonance: prognostic impact of hypo-enhancement evidenced tumor avascularity

**DOI:** 10.1186/s12968-021-00727-2

**Published:** 2021-04-05

**Authors:** Angel T. Chan, William Dinsfriend, Jiwon Kim, Brian Yum, Razia Sultana, Christopher A. Klebanoff, Andrew Plodkowski, Rocio Perez Johnston, Michelle S. Ginsberg, Jennifer Liu, Raymond J. Kim, Richard Steingart, Jonathan W. Weinsaft

**Affiliations:** 1 Department of Medicine, Memorial Sloan Kettering Cancer Center, New York, NY USA; 2 Department of Radiology, Memorial Sloan Kettering Cancer Center, New York, NY USA; 3 Department of Pharmacological Sciences, Icahn School of Medicine At Mount Sinai, New York, NY USA; 4 Department of Medicine, Weill Cornell Medical College, New York, NY USA; 5 Duke Cardiovascular Magnetic Resonance Center, Durham, NC USA

**Keywords:** Cardiovascular magnetic resonance, Cardio-oncology, Cardiac neoplasm

## Abstract

**Background:**

Late gadolinium enhancement (LGE) cardiovascular magnetic resonance (CMR) is widely used to identify cardiac neoplasms, for which diagnosis is predicated on enhancement stemming from lesion vascularity: Impact of contrast-enhancement pattern on clinical outcomes is unknown. The objective of this study was to determine whether cardiac metastasis (C_MET_) enhancement pattern on LGE-CMR impacts prognosis, with focus on heterogeneous lesion enhancement as a marker of tumor avascularity.

**Methods:**

Advanced (stage IV) systemic cancer patients with and without C_MET_ matched (1:1) by cancer etiology underwent a standardized CMR protocol. C_MET_ was identified via established LGE-CMR criteria based on lesion enhancement; enhancement pattern was further classified as heterogeneous (enhancing and non-enhancing components) or diffuse and assessed via quantitative (contrast-to-noise ratio (CNR); signal-to-noise ratio (SNR)) analyses. Embolic events and mortality were tested in relation to lesion location and contrast-enhancement pattern.

**Results:**

224 patients were studied, including 112 patients with C_MET_ and unaffected (C_MET_ -) controls matched for systemic cancer etiology/stage. C_MET_ enhancement pattern varied (53% heterogeneous, 47% diffuse). Quantitative analyses were consistent with lesion classification; CNR was higher and SNR lower in heterogeneously enhancing C_MET_ (p < 0.001)—paralleled by larger size based on linear dimensions (p < 0.05). Contrast-enhancement pattern did not vary based on lesion location (p = NS). Embolic events were similar between patients with diffuse and heterogeneous lesions (p = NS) but varied by location: Patients with right-sided lesions had threefold more pulmonary emboli (20% vs. 6%, p = 0.02); those with left-sided lesions had lower rates equivalent to controls (4% vs. 5%, p = 1.00). Mortality was higher among patients with C_MET_ (hazard ratio [HR] = 1.64 [CI 1.17–2.29], p = 0.004) compared to controls, but varied by contrast-enhancement pattern: Diffusely enhancing C_MET_ had equivalent mortality to controls (p = 0.21) whereas prognosis was worse with heterogeneous C_MET_ (p = 0.005) and more strongly predicted by heterogeneous enhancement (HR = 1.97 [CI 1.23–3.15], p = 0.005) than lesion size (HR = 1.11 per 10 cm [CI 0.53–2.33], p = 0.79).

**Conclusions:**

Contrast-enhancement pattern and location of C_MET_ on CMR impacts prognosis. Embolic events vary by C_MET_ location, with likelihood of PE greatest with right-sided lesions. Heterogeneous enhancement—a marker of tumor avascularity on LGE-CMR—is a novel marker of increased mortality risk.

**Supplementary Information:**

The online version contains supplementary material available at 10.1186/s12968-021-00727-2.

## Background

Nearly 17 million Americans are living with cancer [1], among whom cardiac metastases (C_MET_)bear a major impact on therapeutic decision-making and prognosis. Survival has markedly improved for patients with advanced (stage IV) cancer, resulting in a growing population at risk for C_MET_ and its serious consequences. Data from our group and others have shown C_MET_ to be common with advanced cancer, occurring in up to 20% of patients. [2–6] Embolic events—which can occur when lesions dislodge from the heart—are a leading source of morbidity and mortality among patients with C_MET_. Given that a growing array of new therapies and anticoagulants are available to potentially reduce risk, improved strategies to guide therapy and refine prognostic risk stratification for patients at risk for C_MET_ are of substantial importance.

Cardiovascular magnetic resonance (CMR) has been well-validated for tissue characterization of cardiac masses. [2, 4, 7–12] Whereas neoplasms can vary in morphology, vascular supply is an intrinsic requirement for tumor growth and this property can be leveraged for diagnostic purposes. Using the technique of late gadolinium enhancement (LGE), CMR can identify neoplasms based on vascularity as manifested by *presence* of contrast-enhancement. [13] It is also known that neoplasms can vary in *pattern* of contrast enhancement on LGE-CMR, and that some lesions can include enhancing and non-enhancing components. [2–4] Consistent with this, pathology studies have shown that some neoplasms can have avascular foci (“tumor necrosis”)—a finding linked to aggressive tumor growth and adverse outcomes. [14] Impact of tumor avascularity—as manifested by contrast hypo-enhancement on LGE-CMR—has yet to be tested as a prognostic marker among patients with C_MET_.

This study encompassed a broad cohort of systemic cancer patients with C_MET_ as well as controls (without C_MET_) matched for cancer etiology and stage. CMR was performed using a tailored protocol to assess presence and pattern of C_MET_ enhancement—including standardized grading and quantitative analyses—as well as standardized assessment of lesion size and mobility. The goal was to test impact of C_MET_ anatomic distribution and contrast enhancement pattern on embolic events and mortality.

## Methods

### Study population

The population was comprised of adults (≥ 18 years) with advanced (stage IV) systemic cancer and C_MET_, and controls without C_MET_, matched (1:1) for cancer diagnosis: Presence or absence of C_MET_ was established using the reference of LGE-CMR, on which it was defined via established criteria as a discrete tissue prominence with vascularity as evidenced by contrast-enhancement. [2–4] Subjects with CMR-evidenced intracardiac thrombi were excluded.

**Fig. 1:**
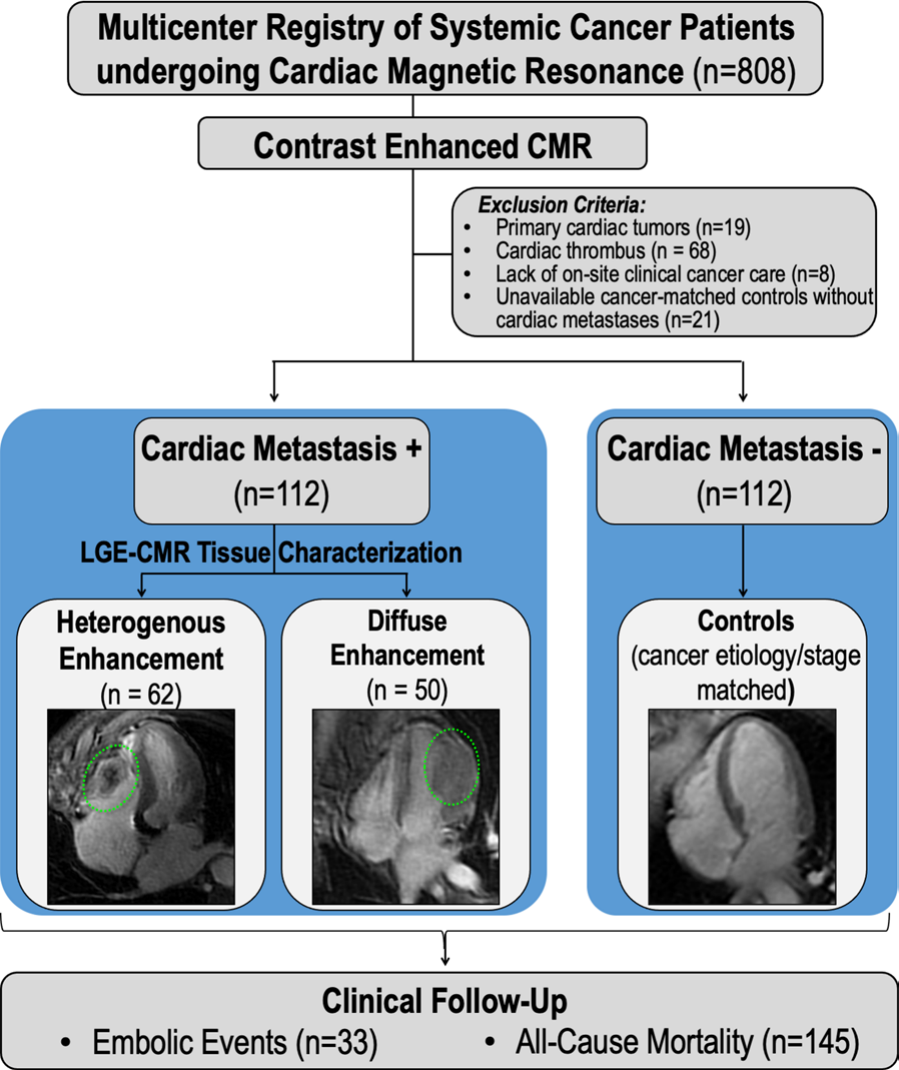
Study design. Overall schematic of multicenter enrollment as well as standardized cardiovascular magnetic resonance (CMR) acquisition and analysis. Note that both enrolling sites employed a tailored CMR protocol for assessment of C_MET_, inclusive dedicated long inversion time (TI) late gadolinium enhancement (LGE)-CMR for evaluation of contrast-enhancement pattern within lesions. Ancillary clinical data were collected in a uniform manner, including baseline cancer-related indices, embolic events, and mortality following CMR

Figure 1 provides a schematic of the research protocol. As shown, study participants were accrued at two tertiary care centers with dedicated cancer care programs (Memorial Sloan Kettering Cancer Center [MSKCC], Weill Cornell Medicine—New York Presbyterian Hospital, New York, New York, USA) that share an integrated CMR program. Clinical data were collected in a standardized manner, including cancer diagnosis and stage, anti-cancer and anticoagulant therapies, as well as clinically documented embolic events (pulmonary embolism (PE), cerebrovascular events (CVA), systemic [splenic, peripheral] emboli) within 6 months of CMR. Mortality was assessed to test prognosis in relation to anatomic and tissue characteristics of C_MET_.

This study entailed analysis of data acquired for clinical purposes between 2012 and 2020; no dedicated interventions were performed for research purposes. Ethics approval for this protocol was provided by the MSKCC and Weill Cornell Medicine institutional review boards, each of which approved a waiver of informed consent for analysis of pre-existing clinical data.

### Imaging protocol

CMR was performed on commercial (1.5 T [87%], 3 T [13%]) scanners (General Electric Healthcare, Waukesha, Wisconsin, USA). Exams included electrocardiogram (ECG)-gated cine- and LGE components; both were obtained in contiguous left ventricular (LV) short (mitral annulus—apex) and long axis (2, 3, and 4 chamber) orientations. Cine-CMR utilized a balanced steady-state free precession (bSSFP) pulse sequence. LGE-CMR utilized an inversion recovery pulse sequence; images were acquired after gadolinium (0.2 mmol/kg) infusion (“long-TI” [600 ms] = 5–10 min, conventional [~ 300 ms] 10–30 min post contrast). Contrast administration entailed gadoterate meglumine (Dotarem, Guerbet, Villepinte, France) or gadopentetate dimeglumine (Magnevist, Bayer Schering Pharma, Berlin, Germany), which were respectively utilized in 53% and 47% of exams. Conventional and long inversion time (TI) LGE-CMR were used to discern presence and pattern of enhancement in C_MET_, concordant with prior methods validated by our group and others. [2–4, 9–11] Conventional LGE-CMR was acquired in all patients; additional breath holds for long TI imaging were tolerated in 92% (103/112) of patients with C_MET_.

### Image analysis

C_MET_ was identified on LGE-CMR based on lesion-associated vascularity in accordance with established qualitative (visual) criteria. [2–4] To test modifying impact of C_MET_ tissue properties on cancer associated outcomes, lesions were further classified into two distinct categories—*diffuse enhancement* (homogenous contrast enhancement throughout lesion) or *heterogeneous enhancement* (enhancing and non-enhancing components within a lesion).

**Fig. 2:**
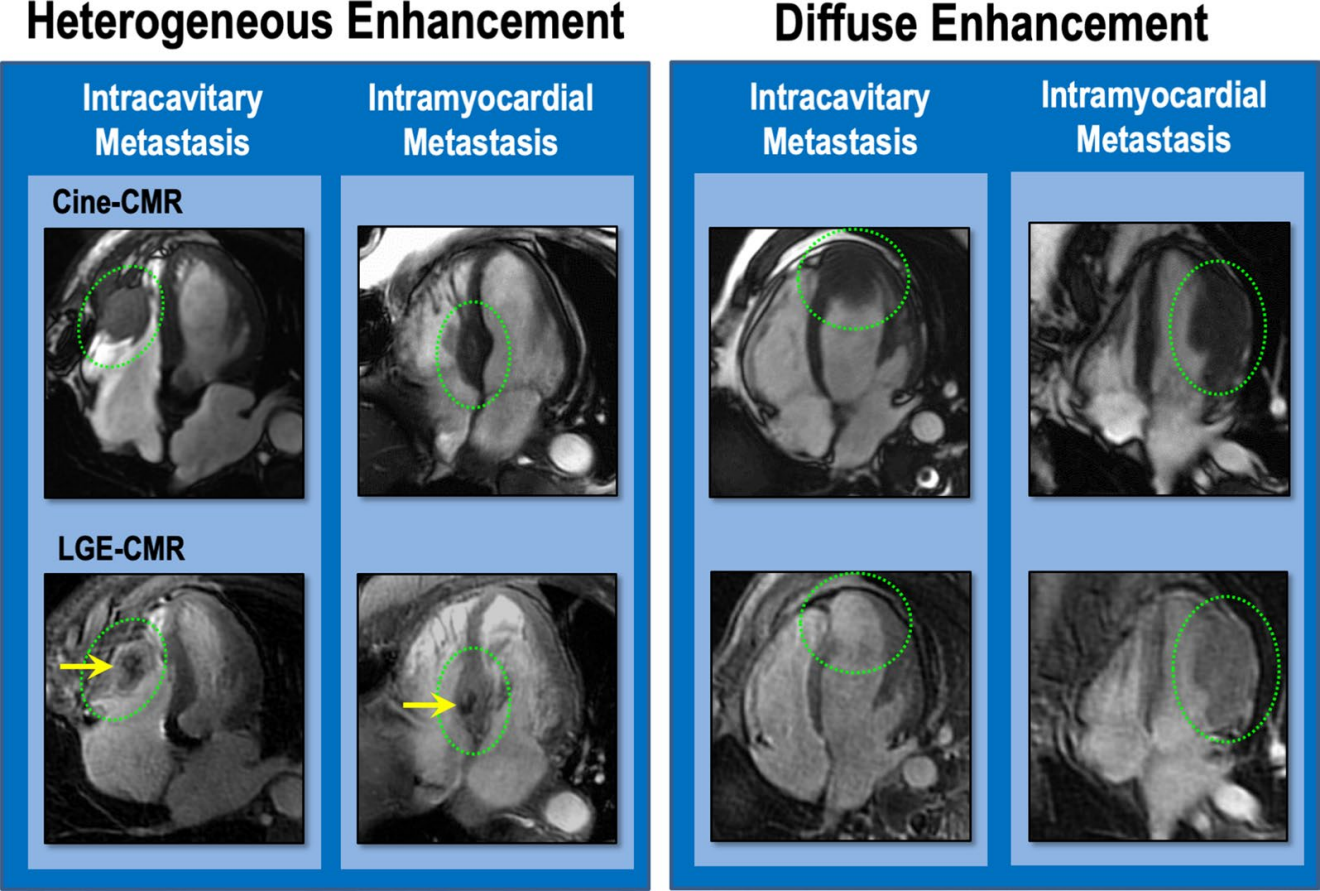
Representative examples. Representative examples of C_MET_ classifications, including heterogeneously (left) and diffusely (right) enhancing lesions with intracavitary or intramural location [top: cine-CMR ∣ bottom: LGE-CMR). Lesions denoted by green circles. Note focal hypo-enhancement (yellow arrows) within heterogeneously enhancing lesions, corresponding to C_MET_ avascular components

C_MET_ were scored in a binary manner (present/absent), localized based on chamber (right atrium [RA], right ventricle [RV], left atrium [LA], left ventricle [LV]) or pericardial involvement, and classified as intra-cavitary (predominantly localized to cardiac chamber) or intramural (invading into myocardium). Intra-cavitary lesions were further graded for lesion mobility, with highly mobile lesions classified as demonstrating dys-synchronous mobility in relation to adjacent myocardium. Figure 2 provides representative examples of C_MET_ types, including heterogeneous and diffusely enhancing lesions with intra-cavitary or intramyocardial involvement.

Quantitative analyses were used to assess magnitude of contrast enhancement within C_MET_. Concordant with established methods used by our group, [2, 4] aggregate signal-to-noise ratio (SNR) and contrast-to-noise ratio (CNR) ratios were measured on the long TI LGE-CMR image on which the lesion was most prominent. Intra-cardiac lesion size (area, linear dimensions) was measured on cine-CMR datasets, which were co-localized with LGE-CMR for purpose of analyses. For patients with multiple C_MET_, the largest lesion was used for quantitative analysis and patient categorization (i.e. heterogeneous or diffusely enhancing). Ancillary analyses included quantification of cardiac chamber size and function, which were measured on cine-CMR using standard planimetry methods. [3]

### Prognostic assessment

Electronic medical records were reviewed to assess embolic events (PE, CVA, systemic [i.e. splenic, peripheral] emboli) within 6 months of imaging, as well as all-cause mortality after CMR so as to test clinical events in relation to presence and type of C_MET_. All-cause mortality and embolic events were ascertained blinded to CMR analyses.

### Statistical methods

Comparisons between groups with or without C_MET_, and between C_MET_ subtypes (heterogeneous, diffusely enhancing) were made using Student’s t-tests (expressed as mean ± standard deviation) for continuous variables, and Chi-square or Fisher’s exact tests for categorical variables: Paired testing (t-tests or McNemar’s tests) was employed for matched case–control comparisons. Univariate logistic regression was used to test variables associated with mortality and embolic events; variables significantly associated with outcomes in univariate analysis were then then tested together in adjusted models. The Kaplan–Meier method was used to calculate survival; follow-up duration was reported as median with interquartile range (IQR). Cox proportional hazards models compared mortality risk between groups, including prognostic utility of C_MET_ features. Calculations were performed using SPSS (Statistical Package for the Social Sciences, International Business Machines, Inc., Armonk, New York, USA). Two-sided p < 0.05 was deemed indicative of statistical significance.

## Results

### Population characteristics

The population comprised 224 adults with advanced (stage IV) systemic cancer undergoing CMR, including 112 patients with C_MET_ as defined by LGE-CMR, and unaffected (C_MET_ -) controls matched for primary cancer diagnosis and stage.

Table 1 details population characteristics, together with comparisons between cancer patients with C_MET_ and their respective controls. As shown, cancer diagnosis varied among patients with C_MET_: Sarcoma, lung, genitourinary, gastrointestinal cancers, and skin/melanoma comprised the leading primary cancer diagnoses, although the population also included patients with primary cancers not typically associated with C_MET_ (e.g. endocrine, head/neck). Regarding anti-cancer regimen, patients with C_MET_ were more likely to be treated with mediastinal radiation therapy and low molecular weight heparin (both p = 0.01) but were otherwise similar with respect to matched controls. Of note, nearly half (49%) of patients with CMR-evidenced C_MET_ had a subsequent change in anti-cancer medication regimen following CMR; 16% received new mediastinal/chest radiation within 3 months after imaging.

**Table 1:** Population characteristics

	Overall (n = 224)	C_MET+_ (n = 112)	C_MET_- Controls (n = 112)	p^b^
Clinical characteristics				
Age (years)	58 ± 17	57 ± 16	58 ± 18	0.68
Gender (male)	59% (133)	63% (71)	55% (62)	0.23
Body surface area (kg/m^2^)^a^	1.9 ± 0.3	1.9 ± 0.3	1.9 ± 0.3	0.55
Cancer etiologies^b^				
Sarcoma	20% (44)	20% (22)	20% (22)	**–**
Lung	16% (36)	16% (18)	16% (18)	**–**
Genitourinary	15% (34)	15% (17)	15% (17)	**–**
Gastrointestinal	13% (28)	13% (14)	14% (14)	**–**
Skin/melanoma	13% (28)	13% (14)	13% (14)	**–**
Lymphoma	8% (18)	8% (9)	8% (9)	**–**
Endocrine	8% (18)	8% (9)	8% (9)	**–**
Anti-cancer regimen				
Chemotherapy				
Alkylating agent	44% (99)	46% (52)	41% (46)	0.47
Plant alkaloid	31% (70)	32% (36)	30% (34)	0.88
Antitumor antibiotics	21% (48)	21% (23)	23% (26)	0.69
Antimetabolites	31% (70)	35% (39)	27% (30)	0.19
Topoisomerase inhibitors	8% (17)	7% (8)	8% (9)	1.00
Anthracycline	21% (46)	19% (21)	23% (26)	0.41
Monoclonal antibodies				
Tyrosine kinase inhibitors	23% (51)	20% (22)	28% (31)	0.21
Other kinase inhibitors	4% (8)	2% (2)	6% (7)	0.18
Immunotherapy	21% (47)	21% (24)	20% (22)	0.84
Radiation therapy				
Mediastinal radiation	13% (28)	18% (20)	7% (8)	*0.01*
Other radiation	38% (84)	40% (45)	35% (39)	0.49
Anticoagulation therapy				
Overall	22% (50)	28% (31)	17% (19)	0.07
Low molecular weight heparin	24% (53)	32% (36)	16% (18)	*0.01*
Warfarin	3% (6)	4% (4)	2% (2)	0.69
Direct Oral Anticoagulant	15% (34)	18% (20)	13% (14)	0.26
Cardiovascular disease risk factors				
Hypertension	43% (97)	41% (46)	45% (51)	0.59
Hyperlipidemia	31% (69)	28% (31)	34% (38)	0.35
Diabetes mellitus	14% (31)	11% (12)	17% (19)	0.23
Smoking	38% (79)	31% (35)	39% (44)	0.26
Cardiopulmonary disease				
Coronary artery disease	11% (24)	11% (12)	11% (12)	1.00
Atrial fibrillation/flutter	16% (35)	13% (14)	19% (21)	0.27
Pulmonary disease	6% (13)	3% (3)	9% (10)	0.09
Pulmonary hypertension	16% (36)	13% (14)	20% (22)	0.17
Cardiac morphology and function				
Left ventricle				
Ejection fraction (%)	61 ± 11	63 ± 8	59 ± 12	*0.004*
End-diastolic volume (mL)	124 ± 41	114 ± 34	131 ± 43	*0.002*
End-systolic volume (mL)	51 ± 29	42 ± 17	57 ± 32	* < 0.001*
Stroke volume (mL)	73 ± 21	72 ± 22	75 ± 21	0.38
Myocardial mass (gm)	118 ± 49	117 ± 55	116 ± 37	0.83
Right ventricle				
Ejection fraction (%)	54 ± 9	54 ± 9	53 ± 8	0.30
End-diastolic volume (mL)	135 ± 44	130 ± 37	141 ± 50	0.08
End-systolic volume (mL)	64 ± 29	60 ± 24	69 ± 32	*0.03*
Stroke volume (mL)	70 ± 23	70 ± 23	72 ± 23	0.44
Atria				
Left atrial area (cm^2^)	20 ± 6	19 ± 5	21 ± 7	*0.02*
Right atrial area (cm^2^)	19 ± 6	19 ± 6	19 ± 6	0.98
Pericardial effusion	24% (53)	30% (33)	19% (21)	0.07

Comparisons between cancer patients with late gadolinium enhancement (LGE) CMR-evidenced cardiac metastases and cancer-matched controls

^§^Among patients with cardiac metastases, 26% had multiple lesions (median lesion # 2.4 ± 1.0); lesion size: 3.8 ± 2.2 cm

C_MET+_, cardiac metastasis; C_MET-_, no cardiac metastasis

^a^Body surface area

^b^ Additional cancer diagnoses: breast (4%), head/neck (3%), mesothelioma (1%), multiple myeloma with extramedullary involvement (1% [stage IV via Southwest Oncology Group criteria [22]])

Regarding cardiac indices, cancer-matched controls referred for CMR were more likely to have adverse left sided chamber remodeling—as evidenced by lower LV ejection fraction and larger chamber size (both p < 0.01), but groups had similar right sided structural and functional indices (p = NS).

### Cardiac metastasis location in relation to contrast enhancement

Anatomic location of C_MET_ varied (LV 32% │LA 22% │, RV 37%│ RA 30%│multi-chamber involvement 30%): Left and right sided chamber involvement were near equal in prevalence (50%, 58% respectively). Regarding C_MET_ tissue properties, 53% of patients had heterogeneously enhancing lesions (enhancing and non-enhancing components on LGE-CMR), whereas 47% had diffusely enhancing lesions without non-enhancing components.

Table 2 compares heterogeneously enhancing and diffusely enhancing C_MET_. As shown, lesions were similar with respect to anatomic distribution, as evidenced by equivalent patterns of chamber involvement and rates of intra-cavitary location (all p = NS). Regarding lesion size, heterogeneously enhancing lesions were larger, based on linear dimensions (p < 0.05). Quantitative analyses were consistent with lesion classification, as evidenced by higher CNR (reflecting greater differences between enhancing and non-enhancing regions) and lower normalized SNR (reflecting impact of non-enhancing lesions on aggregate lesion signal intensity) in heterogeneously enhancing C_MET_ (both p < 0.001 vs. diffusely enhancing C_MET_).

**Table 2:** Anatomic and tissue properties of cardiac metastases

	Heterogeneously enhancing C_MET+_ (n = 59)	Diffusely enhancing C_MET+_ (n = 53)	p
Clinical characteristics			
Cancer etiologies			
Sarcoma	24% (14)	15% (8)	0.25
Lung	15% (9)	17% (9)	0.80
Genitourinary	19% (11)	11% (6)	0.28
Gastrointestinal	14% (8)	11% (6)	0.72
Skin/Melanoma	7% (4)	19% (10)	0.05
Lymphoma	3% (2)	13% (7)	0.08
Endocrine	10% (6)	6% (3)	0.50
Cardiovascular risk factors			
Hypertension	42% (26)	38% (20)	0.50
Hyperlipidemia	27% (16)	28% (15)	0.89
Diabetes mellitus	10% (6)	11% (6)	0.84
Smoking	24% (14)	40% (21)	0.07
Cardiopulmonary disease			
Coronary artery disease	12% (7)	9% (5)	0.68
Atrial fibrillation/flutter	10% (6)	15% (8)	0.43
Pulmonary disease	3% (2)	2% (1)	1.00
Pulmonary hypertension	15% (9)	9% (5)	0.35
Anatomic properties			
Lesion location			
Left ventricle	36% (21)	28% (15)	0.41
Right ventricle	34% (20)	40% (21)	0.53
Left atrium	20% (12)	25% (13)	0.60
Right atrium	24% (14)	38% (20)	0.11
Pericardium	32% (19)	26% (14)	0.50
Left-sided	53% (31)	47% (25)	0.57
Right-sided	49% (29)	68% (36)	*0.04*
Bilateral	17% (10)	23% (12)	0.45
Multi-chamber	25% (15)	36% (19)	0.23
Intra-cavitary			
Left ventricle	9% (5)	8% (4)	1.00
Right ventricle	22% (13)	25% (13)	0.76
Left atrium	17% (10)	21% (11)	0.61
Right atrium	17% (10)	26% (14)	0.22
Lesion number (1 | 2 |≥ 3)	78% | 19% | 3%	70% | 19% | 11%	0.26
Lesion size			
Area (cm^2^)	16.0 ± 20.8	10.1 ± 16.0	0.10
Perimeter (cm)	15.3 ± 11.3	13.3 ± 12.9	0.39
Maximal length (cm)	5.1 ± 3.7	3.7 ± 2.8	*0.02*
Orthogonal length (cm)	3.0 ± 2.0	2.4 ± 1.8	0.11
Perimeter/min Length	5.0 ± 1.7	7.3 ± 8.8	0.06
Pericardial effusion	37% (22)	21% (11)	0.06
Tissue properties			
Contrast-to-noise ratio (CNR)	20.5 ± 15.0	7.4 ± 9.1	* < 0.001*
Signal-to-noise ratio (SNR)	33.1 ± 20.1	44.5 ± 41.2	0.08
Blood pool normalized	0.6 ± 0.2	0.8 ± 0.3	* < 0.001*

### Embolic events

Embolic events (within 6 months of CMR) were assessed to test if presence of C_MET_ impacted likelihood of clinical events, and whether this was modified by lesion location or tissue characteristics. A total of 33 embolic events occurred in the study population; events occurred at a median interval of 2 weeks from CMR [IQR 0.5, 9.5 weeks]. As shown in Table 3A, embolic events were over twofold more common among patients with C_MET_ as compared to cancer matched controls (21% vs. 8%, p = 0.006), including increased incidence of PE (13% vs. 5%, p = 0.08): Embolic events occurred at a median interval of 2 weeks from CMR [IQR 0.5, 9.4 weeks].

**Table 3:** Embolic events

A	C_MET+_	C_MET–_	p^†^
Overall			
All embolic events	21% (24)	8% (9)	*0.006*
Pulmonary embolism^*^	13% (14)	5% (6)	0.08
CVA^†^	8% (9)	4% (4)	0.23
Peripheral embolism	5% (5)	0% (–)	–
Right-sided involvement (n = 65)			
All embolic events	31% (20)	8% (5)	*0.001*
Pulmonary embolism	20% (13)	6% (4)	*0.02*
CVA	9% (6)	3% (2)	0.22
Peripheral embolism	8% (5)	0% (–)	–
Left-sided involvement (n = 56)			
All embolism	13% (7)	7% (4)	0.55
Pulmonary embolism	4% (2)	5% (3)	1.00
CVA	7% (4)	2% (1)	0.38
Peripheral embolism	2% (1)	0% (–)	–
Intra-cavitary metastasis			
Right-sided Involvement (n = 45)			
All embolic events	36% (16)	9% (4)	*0.002*
Pulmonary embolism	27% (12)	7% (3)	*0.01*
CVA	11% (5)	4% (2)	0.38
Peripheral embolism	7% (3)	0% (-)	-
Left-sided Involvement (n = 31)			
All embolic events	29% (9)	10% (3)	0.15
Pulmonary embolism	16% (5)	7% (2)	0.45
CVA	13% (4)	3% (1)	0.38
Peripheral embolism	3% (1)	0% (-)	-

B	Heterogeneous enhancement C_MET+_ (n = 59)	Diffuse enhancement C_MET+_ (n = 53)	P
All embolic events	24% (14)	19% (10)	0.53
Pulmonary embolism	15% (9)	9% (5)	0.35
CVA	10% (6)	6% (3)	0.50
Peripheral embolism	2% (1)	8% (4)	0.19

^*^Pulmonary embolism

^†^Cerebrovascular events

Data shown in Table 3A also demonstrates that C_MET_ location modified likelihood of clinical events: Whereas patients with right sided lesions had a more than threefold increase in PE (20% vs. 6%, p = 0.02), those with left sided lesions had near identical rates of PE compared to cancer-matched controls (4% vs. 5%). Among patients with left sided lesions, CVA was more common compared to matched controls, although statistical differences between groups was not achieved in context of low clinical event rates (7% vs. 2%, p = 0.38). Sub-group analyses limited to intracavitary C_MET_ demonstrated a stronger association between lesion location and embolic event rates: Among patients with right sided intracavitary C_MET_, PE occurred in over one fourth of cases–a rate more than fourfold higher than in matched controls (27% vs. 7%, p = 0.01). Two-thirds (8/12; 67%) of patients with right sided intracavitary C_MET_ who developed PE had lesions graded as highly mobile on cine-CMR.

Notably, increased PE rates among patients with right sided C_MET_ occurred despite frequent anticoagulation: Anticoagulant therapies (warfarin or direct oral anticoagulants) were more commonly utilized in patients with right sided C_MET_ as compared to matched controls (33% vs. 15%, p = 0.02), but were equivalent when among C_MET_ patients with left sided involvement and controls (16% vs. 21%, p = 0.63). Of note, 60% of patients with PE were on anticoagulation at the time of their clinical event (64% in C_MET_ vs 50% in controls, p = 0.64).

**Fig. 3:**
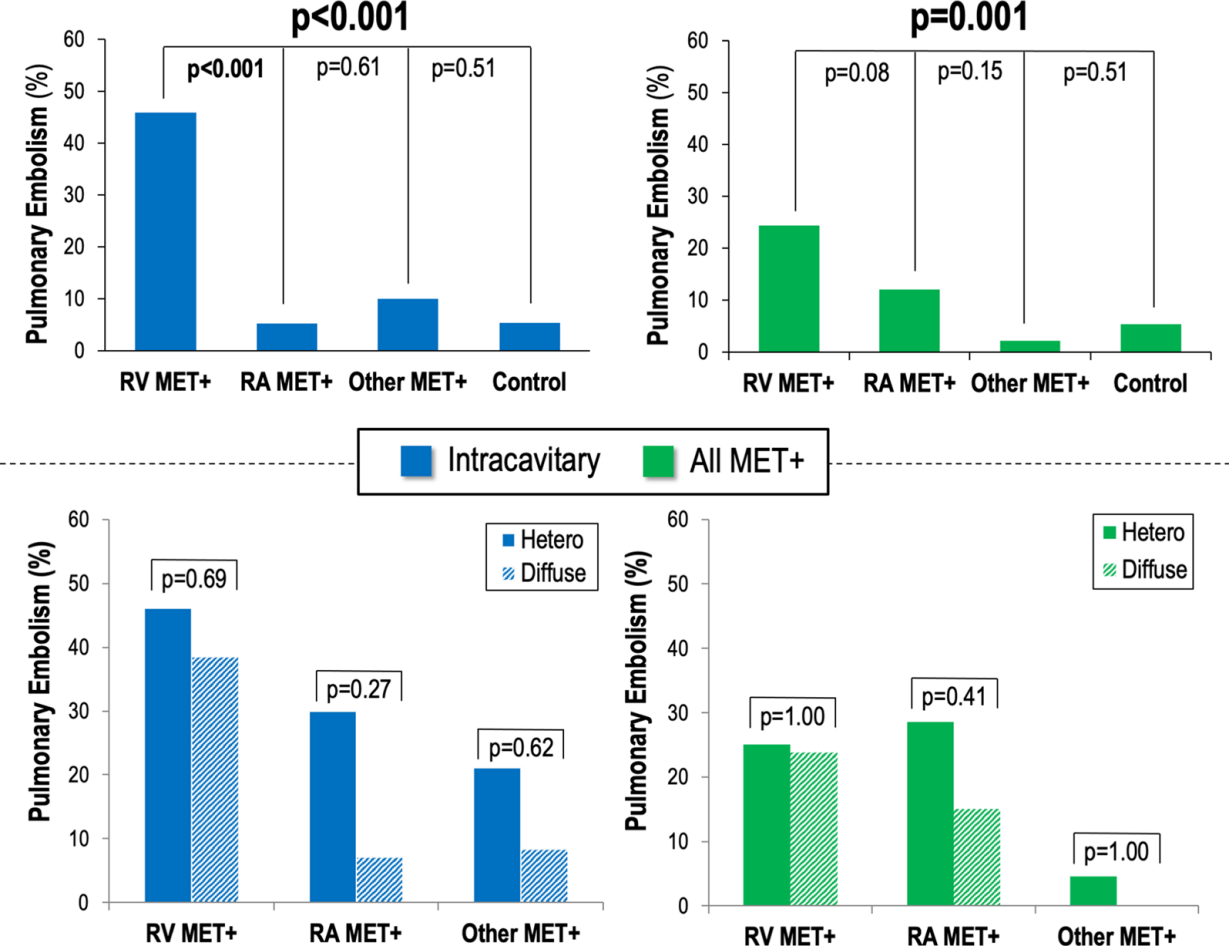
Pulmonary Embolism (PE) in Relation to C_MET_ Location and Tissue Properties. Top: Clinically documented PE (within 6 months of CMR) among patients grouped based on presence and location of C_MET_. Note higher rate of PE among patients with C_MET_ involving the right ventricle, with differences most marked in analysis limited to intra-cavitary lesions (p < 0.001). Bottom: Location-based comparisons of PE rates among patients with heterogeneous and diffusely enhancing C_MET_. Note equivalent rates of PE between patients grouped based on C_MET_ contrast enhancement pattern

Regarding impact of C_MET_ tissue characteristics on embolic events, Table 3B demonstrates that rates of PE were similar between patients with heterogeneous and diffusely enhancing lesions (p = NS), as were rates of left sided embolic events. Figure 3 reports PE rates among patients stratified by both lesion location and tissue characteristics: As shown, PE rates were highest among patients with intracavitary right ventricular lesions (p < 0.001 vs. other groups), whereas partitioning based on lesion tissue characteristics did not stratify event risk (p = NS). **Table ****4** demonstrates that increased PE risk among patients with right ventricular C_MET_ was accompanied by impaired RV function, as evidenced by lower absolute RV ejection fraction (RVEF) and higher prevalence of RV dysfunction (both p < 0.05). Of note, among patients with RV C_MET_, RVEF was similar between those who had PE prior to, compared to those who had PE after CMR (51.0 ± 12.7% vs 53.5 ± 3.8%, p = 0.72)–consistent with the notion that event driven changes in RV systolic function were not responsible for observed associations between impaired RV function and PE. Data shown in Additional file 1: Table S1 tests both clinical and CMR parameters in relation to PE. As shown, both gastrointestinal cancer etiology and right sided C_MET_ were each associated with increased likelihood of PE in univariate regression analysis, and each parameter remained associated with PE (p < 0.01) when the two parameters (gastrointestinal cancer etiology, right sided intracavitary C_MET_) were tested together in an adjusted model.

**Table 4:** Cardiac remodeling in patients with and without right ventricular cardiac metastases

	Right ventricular C_MET+_ (n = 41)	Other C_MET+_ (n = 71)*	p
Cardiac morphology			
Left ventricle			
Ejection fraction (%)	62 ± 8	64 ± 9	0.27
Ejection fraction (< 50%)	10% (4)	9% (6)	1.00
End-diastolic volume (mL)	110 ± 33	117 ± 35	0.27
End-systolic volume (mL)	42 ± 17	42 ± 17	0.83
Stroke volume (mL)	68 ± 21	75 ± 23	0.12
Myocardial mass (gm)	130 ± 81	112 ± 37	0.19
Right ventricle			
Ejection fraction (%)	52 ± 11	56 ± 8	*0.048*
Ejection fraction (< 50%)	34% (14)	16% (11)	*0.03*
End-diastolic volume (mL)	133 ± 41	129 ± 36	0.60
End-systolic volume (mL)	65 ± 30	57 ± 20	0.11
Stroke volume (mL)	68 ± 23	71 ± 23	0.54
Atria			
Left atrial area (cm^2^)	18.2 ± 5.7	19.5 ± 5.1	0.25
Right atrial area (cm^2^)	19.8 ± 6.7	18.7 ± 5.0	0.36
Lesion characteristics			
Anatomic properties			
Area (cm^2^)	9.5 ± 12.4	13.3 ± 19.7	0.27
Perimeter (cm)	14.3 ± 14.0	13.6 ± 10.5	0.79
Maximal Length (cm)	4.1 ± 2.7	4.6 ± 3.9	0.43
Orthogonal Length (cm)	2.3 ± 1.5	2.9 ± 2.2	0.09
Perimeter/Min Length	7.2 ± 8.3	5.6 ± 4.7	0.18
Tissue properties			
Heterogenous Enhancement	49% (20)	55% (39)	0.53
Contrast to Noise Ratio (CNR)	13.5 ± 14.8	14.8 ± 13.8	0.64
Signal-to-Noise Ratio (SNR)	46.8 ± 46.9	33.5 ± 17.0	0.10
Blood pool normalized	0.7 ± 0.3	0.6 ± 0.3	*0.007*

^*^ Inclusive of left ventricular, left atrial, and right atrial cardiac metastases

### Mortality

**Fig. 4:**
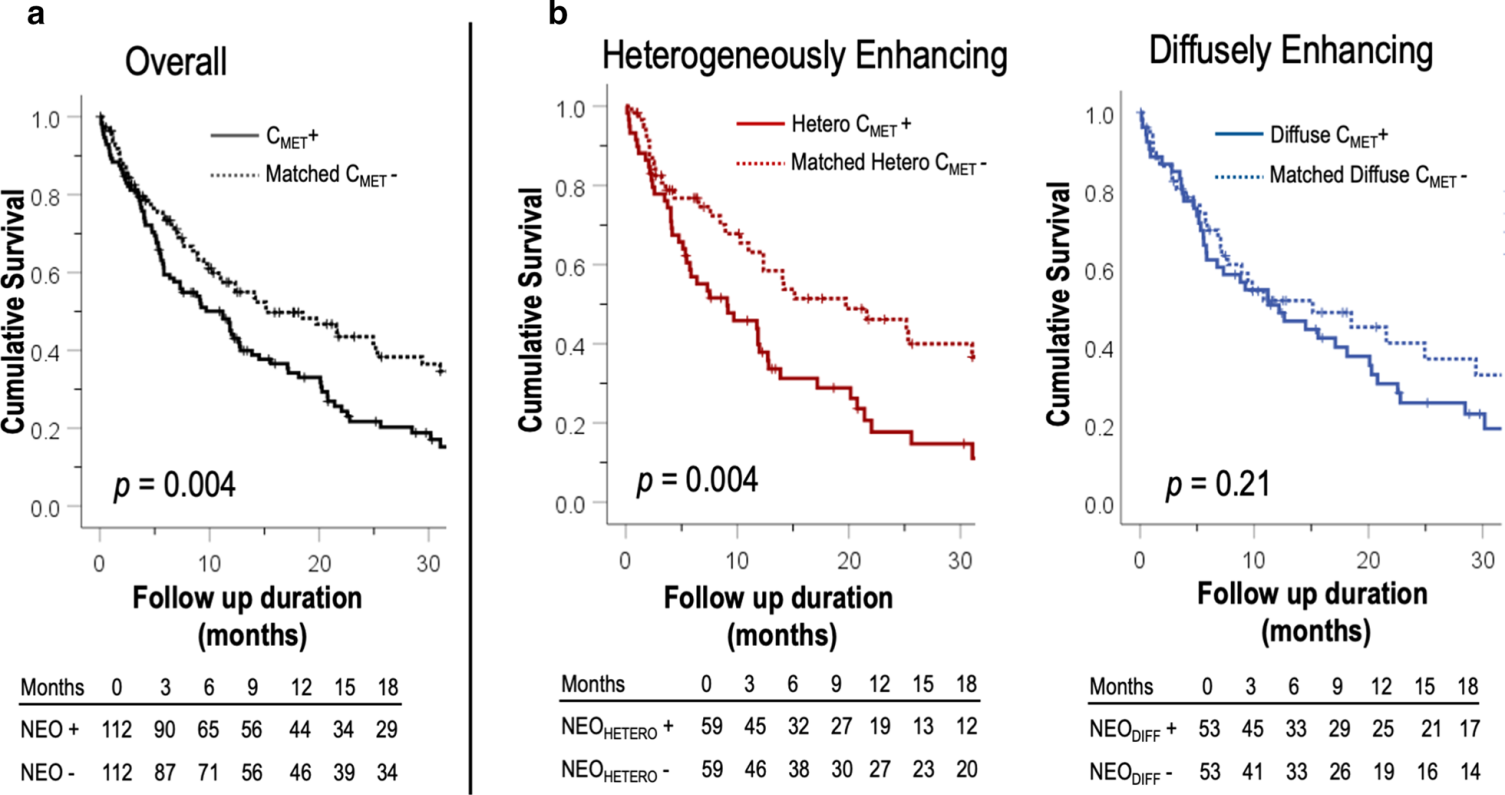
Mortality status. **a** Kaplan Meier survival curves for patients with C_MET_ (solid line) and cancer-matched controls (dotted line), demonstrating increased mortality among patients with C_MET_ compared to (C_MET_ -) controls matched for primary cancer type and stage (p = 0.004). **b** Kaplan–Meier curves among sub-groups with heterogeneously (left) and diffusely enhancing (right) C_MET_ as compared to respective cancer-matched controls. Note that prognosis varied based on C_MET_ tissue properties, as evidenced by equivalent mortality risk between diffusely enhancing C_MET_ and controls (p = 0.21) but increased mortality for patients with heterogeneously enhancing lesions (p = 0.004)

Follow-up was performed for a median duration of 0.8 years [IQR 0.3–1.67], during which a total of 145 deaths occured. Figure 4 provides Kaplan Meier survival curves for the overall cohort of C_MET_ patients and cancer-matched controls, as well as for subgroups based on C_MET_ tissue characteristics. As shown (a), mortality risk was higher among C_MET_ patients compared to controls (HR = 1.64 [CI 1.17–2.29], p = 0.004): Median survival after CMR was shorter among patients with C_MET_ (9.7 months [IQR 4.0–21.7] vs. 15.1 months [5.4–60.3], p = 0.004), paralleled by increased 6-month (39% vs. 28%, p = 0.12) and 1 year (57% vs. 46%, p = 0.2) mortality.

Figure 4b demonstrates that mortality differed in relation to tissue characteristics of C_MET_: Whereas patients with diffusely enhancing C_MET_ had near equivalent mortality to matched controls (p = 0.21), prognosis was worse among patients with heterogeneously enhancing C_MET_ (p = 0.005)—including increased 6-month (44% vs. 26%) and 1 year (65% vs. 41%) mortality in respective case–control comparisons (both p < 0.05). As shown in Table 5, heterogeneously enhancing C_MET_ conferred higher risk for mortality (HR 1.97 [CI 1.23–3.15], p = 0.005) than did number of lesions (HR 1.67 [CI 1.31–2.12], p < 0.001) or lesion size (1.11 per 10 cm [CI 0.53–2.33], p = 0.79). Additionally, whereas lymphoma was the sole cancer diagnosis associated with differential (improved) prognosis, an adjusted model analysis inclusive of this variable together with LGE-CMR tissue characterization data demonstrated heterogeneously enhancing C_MET_ to remain associated with increased mortality (1.97 [CI 1.23–3.16], p = 0.005).

**Table 5:** Mortality predictors

	Hazard ratios	p
Clinical characteristics		
Age (years)	1.00 [CI 1.00–1.01]	0.36
Gender (male)	1.25 [CI 0.88–1.76]	0.20
Cancer etiologies		
Sarcoma	0.91 [CI 0.61–1.37]	0.65
Lung	1.45 [CI 0.95–2.21]	0.08
Genitourinary	1.12 [CI 0.73–1.73]	0.59
Gastrointestinal	1.56 [CI 0.96–2.54]	0.07
Skin/melanoma	0.71 [CI 0.41–1.20]	0.20
Lymphoma	0.40 [CI 0.19–0.86]	*0.02*
Endocrine	0.62 [CI 0.31–1.22]	0.16
Cardiovascular risk factors		
Hypertension	0.98 [CI 0.70–1.35]	0.86
Hyperlipidemia	1.00 [CI 0.70–1.44]	0.99
Diabetes mellitus	0.87 [CI 0.52–1.46]	0.59
Smoking	1.01 [CI 0.71–1.41]	0.98
Cardiopulmonary disease		
Coronary artery disease	1.36 [CI 0.84–2.21]	0.21
Atrial fibrillation/flutter	1.16 [CI 0.74–1.82]	0.52
Pulmonary disease	0.86 [CI 0.41–1.77]	0.67
Pulmonary hypertension	1.33 [CI 0.86–2.06]	0.20
Cardiac morphology		
Left ventricle		
Ejection fraction	1.01 [CI 0.99–1.03]	0.21
Ejection fraction (< 50%)	0.82 [CI 0.50–1.36]	0.44
End-diastolic volume	1.00 [CI 0.99–1.00]	0.06
End-systolic volume	0.99 [CI 0.99–1.00]	0.07
Right ventricle		
Ejection fraction	1.02 [CI 1.00–1.04]	0.09
Ejection fraction (< 50%)	0.84 [CI 0.57–1.26]	0.40
C_MET_ Lesion Characteristics		
C_MET_ (presence vs. absence)^*^	1.64 [CI 1.17–2.29]	*0.004*
Anatomic properties		
Lesion number	1.67 [CI 1.31–2.12]	* < 0.001*
Multiple lesions	1.94 [CI 1.23–3.06]	*0.004*
Lesion size (maximal diameter [per 10 cm])	1.11 [CI 0.53–2.33]	0.79
Lesion size (area [per 10 cm^2^])	0.99 [CI 0.85–1.15]	0.88
Intra-cavitary lesion	1.27 [CI 0.81–1.97]	0.30
Tissue properties^*^		
Heterogeneous enhancement	1.97 [CI 1.23–3.15]	*0.005*
Diffuse enhancement	1.36 [CI 0.84–2.21]	0.21
Adjusted multivariate model^†^		
Lymphoma (cancer etiology)	0.44 [CI 0.11–1.79]	0.24
Heterogeneous enhancement	1.97 [CI 1.23–3.16]	*0.005*

^*^ Comparison between cancer patients with CMR-evidenced cardiac metastases and cancer-matched controls

^†^ Regression analysis performed incorporating lymphoma (sole cancer associated with differential [improved] prognosis) and C_MET_ heterogeneous enhancement together in an adjusted model (no additional variables included in adjusted models)

## Discussion

To our knowledge, this is the first study to test LGE-CMR pattern of C_MET_ as a prognostic marker in patients with systemic cancer, with focus on localized hypo-enhancement (a marker of tumor avascularity) as a novel marker of adverse prognosis. Results add to a growing body of literature by our group and others validating LGE-CMR in relation to histopathology and demonstrating clinical utility of this approach to guide diagnostic, prognostic, and therapeutic decision-making for patients with known or suspected cardiac masses. Key findings are as follows: First, among a broad cohort of advanced cancer patients, C_MET_ contrast-enhancement pattern varied—prevalence of diffusely (47%) and heterogeneously enhancing (53%) lesions was near equivalent. Quantitative analyses demonstrated heterogeneously enhancing C_MET_ to have more aggressive features, as evidenced by larger lesion size and lower SNR (as would be expected in context of tumor avascularity). Second, presence and distribution of C_MET_ impacted likelihood of embolic events. Aggregate embolic events were higher among patients with C_MET_ compared to cancer matched controls (21% vs. 8%, p = 0.006), C_MET_ location modified likelihood of events: Whereas patients with right sided lesions had a threefold increase in PE (20% vs. 6%, p = 0.02), those with left sided lesions had near identical event rates to those of (C_MET_ -) controls (4% vs. 5% p = 1.00). Embolic event rates did not vary in relation to C_MET_ by tissue properties, as evidenced by equivalent rates of PE among patients with diffuse and heterogeneously enhancing right ventricular lesions. Third, mortality risk conferred by C_MET_ varied in relation to contrast-enhancement pattern. During a median follow-up of 0.7 years [IQR 0.3–1.7], patients with and without diffusely enhancing C_MET_ had equivalent mortality to controls (p = 0.21), whereas prognosis was worse among patients with heterogeneously enhancing C_MET_ compared to controls matched for cancer etiology and stage (p = 0.005).

Our finding that heterogeneous lesion enhancement constitutes an adverse prognostic marker among patients with C_MET_ is consistent with established concepts in tumor biology: Tumor necrosis—as would be expected to result in avascularity and thus impaired contrast uptake—is a known marker of aggressive phenotype: Uncontrolled oncogene driven proliferation of neoplastic cells exhausts oxygen supply from normal vasculature, resulting in localized hypoxia which upregulates production of angiogenic factors and triggers neovascularization. [15, 16] However, vessels formed in response to hypoxia lack normal physiological angiogenesis—providing a nidus for chaotic tumor architecture and vascular leakiness. Hypoxia alters cancer metabolism to foster survival during stress and drive malignant progression, resulting in resistance to anti-cancer therapy and accelerated tumor growth. Consistent with this, magnetic resonance imaging (MRI) studies focused on extra-cardiac areas—including neurologic and renal cancers—have associated contrast hypo-enhancement (tumor necrosis) with chemotherapeutic resistance and increased mortality. [14, 17, 18].

Regarding embolic events, our data demonstrated C_MET_ location to be strongly associated with outcomes—as evidenced by increased rates of PE among patients with RV intracavitary C_MET_. Our finding that embolic events were equivalent between patients with diffuse and heterogeneous C_MET_ is not unexpected, given that avascular components were typically centrally located within lesions and would thus be unexpected to provide a nidus for embolization. Regarding mechanism, our data demonstrated patients with RV C_MET_ to have lower RV systolic function than those with C_MET_ in other locations (p < 0.05)—possibly due to mechanical tumor effects or treatment related (i.e. radiation-induced) cardiac injury. Based on this, it is possible that localized stasis could contribute to development of super-imposed thrombosis on neoplastic lesions—providing a nidus for embolic events. Whereas heterogeneous enhancement (as would be expected in context of tumor with superimposed thrombus) was not identified as a risk factor for embolic events, it is possible that micro-thrombi developed prior to or following the time of CMR, or that spatial resolution of LGE-CMR was insufficient for diagnostic detection. It is also possible that emboli stemmed from tumor dislodgement rather than primary thrombotic processes or from insufficient anticoagulation—concepts supported by the fact that nearly two-thirds (63%) of C_MET_ patients were on anticoagulation at the time of clinical events, as well as recent data showing high embolic event rates in non-cancer [10, 19] and cancer populations [20] with cardiac thrombus treated with anticoagulants. Future research, including imaging using high resolution 3D LGE-CMR [21] and prospective trials testing relative efficacy of anticoagulant regimens or targeted resection in cancer patients with C_MET_, are necessary to further test these concepts.

### Limitations

Several limitations should be noted. First, whereas our study encompassed a broad cohort of cancer patients undergoing CMR and clinical follow-up at two institutions, it should be recognized that embolic events were ascertained based on clinical documentation and/or diagnostic testing. In this context, it is likely that subtle clinical events were under-reported, or that clinical considerations may have influenced testing such that embolic events such as stroke were under-diagnosed. It should be noted that our study was unable to reliably ascertain all potential cancer related clinical indices or derive aggregate classifications of disease chronicity and performance status. Thus, while our results demonstrate that location and contrast-enhancement pattern of LGE-CMR evidenced C_MET_ impacts clinical outcomes, further research is warranted to test whether associations observed in the current study are modified by cancer etiology, treatment type, and/or overall health status. Second, whereas our study utilized LGE-CMR for tissue characterization of C_MET_, alternative approaches to assess vascularity such as quantitative LGE thresholds, perfusion, parametric mapping, or susceptibility weighted CMR were not tested. Whereas current findings demonstrate heterogeneous contrast uptake pattern within C_MET_ to be a marker of increased mortality risk, knowledge gaps persist as to the relative utility of different imaging approaches to assess tumor necrosis or additional tissue properties such as hemorrhage and calcification. It is also important to recognize that whereas prior studies have validated LGE-CMR tissue characterization of masses in relation to pathology and other standards including metabolic imaging and outcomes, [3, 4, 10] lack of systematic biopsy sampling in the current cohort (for whom invasive cardiac tissue sampling was uncommon in context of advanced cancer) prohibited direct comparison of LGE-CMR enhancement pattern to pathology findings. An additional limitation relates to the relatively small number of embolic events (n = 33) in this study, which may explain the wide confidence intervals with respect to observed associations of C_MET_ and GI cancer etiology with PE: In this context, current results should be considered more exploratory than definitive, thus highlighting the need to test them further in larger scale studies. Future research is also warranted to test whether alternative protocols using low dose contrast administration or non-contrast approaches (e.g. T1 mapping) provide equivalent diagnostic and prognostic utility in cancer patients with known or suspected C_MET_.

## Conclusions

Location and contrast-enhancement pattern of C_MET_ impact clinical outcomes, with RV lesion location associated with PE and heterogeneous enhancement conferring increased mortality. Given current findings, future research is warranted to test anticoagulant strategies in cancer populations, whether adverse prognosis conferred by heterogeneous lesion enhancement stems from accelerated tumor growth, and whether tailored therapies—paired to lesion tissue characteristics—improves clinical outcomes for cancer patients with C_MET_.

## Supplementary Information

**Additional file 1:** Supplementary Table 1.

## Data Availability

Study data can be made available to other researchers for purposes of reproducing the results of this study on request (contingent on approval of the Memorial Sloan Kettering Cancer Center and Weill Cornell institutional review boards and assurance of data de-identification).

## Abbreviations

bSSFP: Balanced steady-state free precession
CI: Confidence interval
C_MET_: Cardiac metastasis
CMR: Cardiovascular magnetic resonance
CNR: Contrast-to-noise ratio
CVA: Cerebrovascular events
ECG: Electrocardiogram
HR: Hazard ratio
IQR: Interquartile range
LA: Left atrium/left atrial
LGE: Late gadolinium enhancement
LV: Left ventricle/left ventricular
MRI: Magnetic resonance imaging
PE: Pulmonary embolism
RA: Right atrium/right atrial
RV: Right ventricle/right ventricular
RVEF: Right ventricular ejection fraction
SNR: Signal-to-noise ratio
TI: Inversion time

## References

[1] National Cancer Institute: Surveillance, Epidemiology, and End Results Program. https://seer.cancer.gov/statistics/. 2019. Accessed 15 Jan 2020.

[2] Chan AT, Plodkowski AJ, Pun SC (2017). Prognostic utility of differential tissue characterization of cardiac neoplasm and thrombus via late gadolinium enhancement cardiovascular magnetic resonance among patients with advanced systemic cancer. J Cardiovasc Magn Reson.

[3] Chan AT, Fox J, Perez Johnston R (2019). Late gadolinium enhancement cardiac magnetic resonance tissue characterization for cancer-associated cardiac masses: metabolic and prognostic manifestations in relation to whole-body positron emission tomography. J Am Heart Assoc.

[4] Pun SC, Plodkowski A, Matasar MJ (2016). Pattern and prognostic implications of cardiac metastases among patients with advanced systemic cancer assessed with cardiac magnetic resonance imaging. J Am Heart Assoc.

[5] Lam KY, Dickens P, Chan AC (1993). Tumors of the heart. A 20-year experience with a review of 12,485 consecutive autopsies. Arch Pathol Lab Med.

[6] Verso M, Agnelli G (2003). Venous thromboembolism associated with long-term use of central venous catheters in cancer patients. J Clin Oncol.

[7] Fussen S, De Boeck BWL, Zellweger MJ (2011). Cardiovascular magnetic resonance imaging for diagnosis and clinical management of suspected cardiac masses and tumours. Eur Heart J.

[8] Srichai MB, Junor C, Rodriguez LL (2006). Clinical, imaging, and pathological characteristics of left ventricular thrombus: a comparison of contrast-enhanced magnetic resonance imaging, transthoracic echocardiography, and transesophageal echocardiography with surgical or pathological validation. Am Heart J.

[9] Weinsaft JW, Kim HW, Crowley AL (2011). LV thrombus detection by routine echocardiography: insights into performance characteristics using delayed enhancement CMR. JACC Cardiovasc Imaging.

[10] Weinsaft JW, Kim HW, Shah DJ (2008). Detection of left ventricular thrombus by delayed-enhancement cardiovascular magnetic resonanceprevalence and markers in patients with systolic dysfunction. J Am Coll Cardiol.

[11] Weinsaft JW, Kim RJ, Ross M (2009). Contrast-enhanced anatomic imaging as compared to contrast-enhanced tissue characterization for detection of left ventricular thrombus. JACC Cardiovasc Imaging.

[12] Mousavi N, Cheezum MK, Aghayev A (2019). Assessment of cardiac masses by cardiac magnetic resonance imaging: histological correlation and clinical outcomes. J Am Heart Assoc.

[13] Pazos-Lopez P, Pozo E, Siqueira ME (2014). Value of CMR for the differential diagnosis of cardiac masses. JACC Cardiovasc Imaging.

[14] Beddy P, Genega EM, Ngo L (2014). Tumor necrosis on magnetic resonance imaging correlates with aggressive histology and disease progression in clear cell renal cell carcinoma. Clin Genitourinary Cancer.

[15] Bergers G, Benjamin LE (2003). Tumorigenesis and the angiogenic switch. Nat Rev Cancer.

[16] Eales KL, Hollinshead KE, Tennant DA (2016). Hypoxia and metabolic adaptation of cancer cells. Oncogenesis.

[17] Hammoud MA, Sawaya R, Shi W, Thall PF, Leeds NE (1996). Prognostic significance of preoperative MRI scans in glioblastoma multiforme. J Neurooncol.

[18] Nowosielski M, Gorlia T, Bromberg JEC (2019). Imaging necrosis during treatment is associated with worse survival in EORTC 26101 study. Neurology.

[19] Lattuca B, Bouziri N, Kerneis M (2020). Antithrombotic therapy for patients with left ventricular mural thrombus. J Am Coll Cardiol.

[20] Plodkowski AJ, Chan A, Gupta D (2020). Diagnostic utility and clinical implication of late gadolinium enhancement cardiac magnetic resonance for detection of catheter associated right atrial thrombus. Clin Imaging.

[21] Nguyen TD, Spincemaille P, Weinsaft JW (2008). A fast navigator-gated 3D sequence for delayed enhancement MRI of the myocardium: comparison with breathhold 2D imaging. J Magn Reson Imaging.

[22] Choi J-H, Yoon J-H, Yang S-K (2007). Clinical value of new staging systems for multiple myeloma. Cancer Res Treat.

