# Supplementary material for: Risk stratification of cardiac metastases using late gadolinium enhancement cardiovascular magnetic resonance: prognostic impact of hypo-enhancement evidenced tumor avascularity
Source: J Cardiovasc Magn Reson. 2021 Apr 5;23:42. doi: 10.1186/s12968-021-00727-2 (PMC8020547; doi:10.1186/s12968-021-00727-2)
Supplement: Supplementary file 1 — Additional file 1: Supplementary Table 1. [file 12968_2021_727_MOESM1_ESM.docx]

**Supplemental Table. Predictors of Pulmonary Embolism**

|  | Odd Ratios | **p** |
| --- | --- | --- |
| **Clinical Characteristics** |  |  |
| Age | 1.01 [CI 0.98 – 1.03] | 0.75 |
| Gender | 0.66 [CI 0.26 – 1.65] | 0.37 |
| Cancer Etiologies |  |  |
| Sarcoma | 0.71 [CI 0.20 – 2.51] | 0.59 |
| Lung | 0.91 [CI 0.25 – 3.30] | 0.89 |
| Genitourinary | 0.99 [CI 0.27 – 3.56] | 0.98 |
| Gastrointestinal | 6.13 [CI 2.24 – 16.78] | **<0.001** |
| Skin/melanoma | 0.76 [CI 0.17 – 3.47] | 0.72 |
| Lymphoma | - | 1.00 |
| Endocrine | 0.58 [CI 0.07 – 4.59] | 0.61 |
| **Cardiovascular Risk Factors** |  |  |
| Hypertension | 0.41 [CI 0.14 – 1.16] | 0.09 |
| Hyperlipidemia | 0.54 [CI 0.17 – 1.66] | 0.28 |
| Diabetes mellitus | 1.11 [CI 0.31 – 4.03] | 0.88 |
| Smoking | 0.99 [CI 0.38 – 2.59] | 0.98 |
| **Cardiopulmonary Disease** |  |  |
| Coronary artery disease | - | 1.00 |
| Pulmonary disease | - | 1.00 |
| Pulmonary hypertension | 1.34 [CI 0.42 – 4.28] | 0.62 |
| **C_MET_ Lesion Characteristics** |  |  |
| C_MET_ (presence vs. absence)**^*^** | 2.52 [CI 0.93 – 6.83] | 0.07 |
| **Anatomic Properties**^‡^ |  |  |
| Lesion number | 0.80 [CI 0.30 – 2.10] | 0.64 |
| Multiple lesions | 1.17 [CI 0.34 – 4.06] | 0.81 |
| Lesion size (maximal diameter [per 10 cm]) | 0.35 [CI 0.001 – 1.16] | 0.06 |
| Lesion size (area [per 10 cm^2^]) | 0.24 [CI 0.05 – 1.13] | 0.07 |
| **Tissue Properties** |  |  |
| Heterogeneous enhancement (vs. diffuse) | 1.73 [CI 0.54 – 5.53] | 0.36 |
| **Location**^†^ |  |  |
| Right sided C_MET_ | 11.50 [CI 1.45 – 91.34] | **0.02** |
| Right sided intra-cavitary C_MET_ | 11.81 [CI 2.50 – 55.93] | **0.002** |
| **Adjusted Model**** |  |  |
| Gastrointestinal cancer etiology | 5.49 [CI 1.84 – 16.35] | **0.002** |
| Right sided intra-cavitary C_MET_ | 7.22 [CI 2.63 – 19.78] | **<0.001** |

***** Comparison between cancer patients with CMR-evidenced cardiac metastases and cancer-matched controls

† Comparison among patients with CMR-evidenced cardiac metastases who had embolism

^‡^ Comparison between cancer patients with CMR-evidenced cardiac metastases only

** Regression analysis performed incorporating gastrointestinal cancer etiology and right sided intra-cavitary C_MET_ together in adjusted model (no additional variables included in adjusted models).
